# Red blood cells capture and deliver bacterial DNA to drive host responses during polymicrobial sepsis

**DOI:** 10.1172/JCI182127

**Published:** 2024-12-12

**Authors:** L.K. Metthew Lam, Nathan J. Klingensmith, Layal Sayegh, Emily Oatman, Joshua S. Jose, Christopher V. Cosgriff, Kaitlyn A. Eckart, John McGinniss, Piyush Ranjan, Matthew Lanza, Nadir Yehya, Nuala J. Meyer, Robert P. Dickson, Nilam S. Mangalmurti

**Affiliations:** 1 Division of Pulmonary, Allergy, and Critical Care and; 2 Division of Traumatology, Surgical Critical Care and Emergency Surgery, Perelman School of Medicine, University of Pennsylvania, Philadelphia, Pennsylvania, USA.; 3 Pulmonary and Critical Care Unit, Department of Medicine, Massachusetts General Hospital, Boston, Massachusetts, USA.; 4 Division of Pulmonary and Critical Care Medicine, Department of Internal Medicine, University of Michigan Health System, Ann Arbor, Michigan, USA.; 5 Department of Comparative Medicine, Penn State Health Milton S. Hershey Medical Center, Hershey, Pennsylvania, USA.; 6 Division of Pediatric Critical Care, Perelman School of Medicine at the University of Pennsylvania, Philadelphia, Pennsylvania, USA.; 7 Institute for Immunology, Perelman School of Medicine, University of Pennsylvania, Philadelphia, Pennsylvania, USA.; 8 Weil Institute for Critical Care Research and Innovation, Ann Arbor, Michigan, USA.; 9 Department of Microbiology and Immunology, University of Michigan Medical School, Ann, Arbor, Michigan, USA.

**Keywords:** Inflammation, Pulmonology, Cytokines, Innate immunity

## Abstract

Red blood cells (RBCs), traditionally recognized for their role in transporting oxygen, play a pivotal role in the body’s immune response by expressing TLR9 and scavenging excess host cell-free DNA. DNA capture by RBCs leads to accelerated RBC clearance and triggers inflammation. Whether RBCs can also acquire microbial DNA during infections is unknown. Murine RBCs acquire microbial DNA in vitro, and bacterial DNA–induced (bDNA-induced) macrophage activation was augmented by WT, but not Tlr9-deleted, RBCs. In a mouse model of polymicrobial sepsis, RBC-bound bDNA was elevated in WT mice but not in erythroid Tlr9–deleted mice. Plasma cytokine analysis in these mice revealed distinct sepsis clusters characterized by persistent hypothermia and hyperinflammation in the most severely affected mice. RBC Tlr9 deletion attenuated plasma and tissue IL-6 production in the most severely affected group. Parallel findings in humans confirmed that RBCs from patients with sepsis harbored more bDNA than did RBCs from healthy individuals. Further analysis through 16S sequencing of RBC-bound DNA illustrated distinct microbial communities, with RBC-bound DNA composition correlating with plasma IL-6 in patients with sepsis. Collectively, these findings unveil RBCs as overlooked reservoirs and couriers of microbial DNA, capable of influencing host inflammatory responses in sepsis.

## Abstract 2

Red blood cells capture microbial DNA, altering the host response during sepsis. Moreover, human RBCs are a reservoir of microbial DNA.

## Introduction

Recognition of microbe-derived nucleic acid by cytosolic and endosomal receptors is vital for initiating inflammatory responses essential for host defense. We have recently discovered that mammalian red blood cells (RBCs) express the nucleic acid–sensing receptor TLR9 and can bind mitochondrial DNA, functioning as immune sentinels by triggering rapid senescence and loss of self-tolerance, with consequent clearance and innate immune activation upon excess binding of DNA (1). Collectively, these findings suggest that circulating RBCs modulate host immune responses during inflammatory states, yet whether RBCs acquire bacterial DNA (bDNA) during sepsis remains unknown.

While elevated plasma cell-free, CpG-containing mitochondrial DNA is a hallmark of critical illness syndromes, including sepsis and trauma, an emerging body of literature has demonstrated elevated bDNA in the circulation during sepsis and pneumonia (2, 3). In light of the accumulating evidence demonstrating the association of circulating microbial DNA and host inflammatory responses and the plausible interaction between RBCs and bacteria in the vascular compartment, we asked whether RBCs could acquire microbial DNA and orchestrate distinct host inflammatory responses (4). In a murine model of polymicrobial sepsis, we show that RBCs captured microbial DNA during sepsis and that RBC Tlr9–mediated DNA delivery drove hyperinflammation. Moreover, RBCs from critically ill patients with sepsis had a distinct microbial DNA composition compared with those from healthy donors, which correlated with the systemic inflammatory response. Thus, we found that the process of RBC-mediated DNA capture and delivery shaped diverse host inflammatory responses during sepsis.

## Results

### Cecal slurry injection recapitulates human sepsis features, and RBC-bound bDNA is elevated following cecal slurry–induced sepsis.

**Figure 1:**
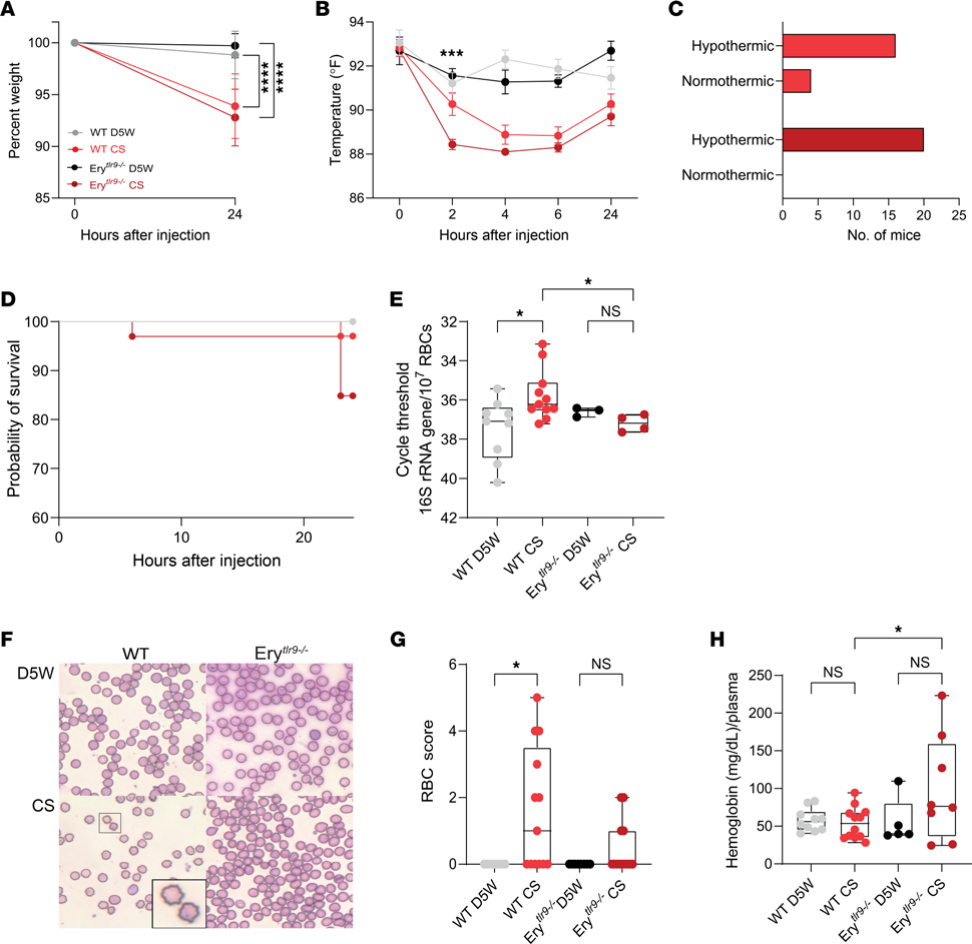
RBCs from WT but not *Ery*^tlr9–/–^ mice acquire microbial DNA and demonstrate morphologic changes during sepsis. Ery*^tlr9–/–^* mice and their WT littermates were injected with CS or D5W and monitored for 24 hours. (**A**) Weight change and differences between groups were analyzed by 1-way ANOVA with Šidák’s multiple-comparison test. ****P < 0.0001 for WT D5W versus WT CS and Ery*^tlr9–/–^* D5W versus Ery*^tlr9–/–^* CS, WT CS versus Ery*^tlr9–/–^* CS = NS. *n* = 20–30 mice per group. (**B**) For temperature profiles of injected mice, 88°F was the lower limit of detection. ANOVA with Šidák’s multiple-comparison analysis, ***P = 0.002 between CS-injected groups at 2 hours, **P = 0.005 between Ery*^tlr9–/–^* D5W and Ery*^tlr9–/–^* CS. *n* = 9–20 mice per group. (**C**) Hypothermic state of the mice by strain at 24 hours. *P* = 0.035, by χ^2^ test. (**D**) Kaplan-Meier survival with log-rank comparison. *P* = 0.031 between all groups; *P* = 0.08 for WT CS-injected versus Ery*^tlr9–/–^* CS-injected mice. (**E**) RBC-associated 16S rRNA gene expression on murine RBCs was quantified 6 hours following CS-induced sepsis. One-way Kruskal-Wallis test with Dunn’s multiple-comparison test. *n* = 3–11. *P = 0.042 WT D5W versus WT CS; *P = 0.044 WT CS versus Ery*^tlr9–/–^* CS. (**F**) RBCs from WT or Ery*^tlr9–/–^* mice 24 hours after D5W or CS injection. Inset shows echinocytic RBCs observed in the WT CS-injected mice. Original magnification, ×40. (**G**) RBC score (number of echinocytes and altered RBCs/high-power field [hpf]). *P = 0.013, WT D5W versus WT CS. *n* = 8–14 from 3 independent studies. (**H**) Cell-free hemoglobin 24 hours after CS injection. Differences between groups were measured using 1-way ANOVA with Šidák’s multiple-comparison test. *P = 0.044 for WT CS versus Ery*^tlr9–/–^* CS; *P* = NS for all other comparisons.

Although no preclinical model can recapitulate all of the features of a clinical syndrome, the cecal slurry (CS) model of polymicrobial sepsis captures the hallmark features of sepsis, including organ dysfunction, temperature disruption, and cytokine storm (5, 6). To investigate the role of RBC DNA capture in sepsis, we induced CS sepsis in WT mice and mice in which Tlr9 was deleted in erythrocytes (Ery*^tlr9–/–^*). The baseline characteristics of the mice have been previously described (1) and are also described in Supplemental Figure 1 (supplemental material available online with this article; https://doi.org/10.1172/JCI182127DS1). Tlr9 expression was retained on immune cells in the Ery*^tlr9–/–^* mice (Supplemental Figure 1, A–G), and we observed no differences in baseline spleen or liver weights or RBC hemoglobin content (Supplemental Figure 1, H–J) (1). CS injection induced weight loss and hypothermia in all mice (Figure 1, A–C). At early time points but not later time points, Ery*^tlr9–/–^* mice had increased hypothermia when compared with WT mice (Figure 1B). Although a subset of WT mice never developed hypothermia, all Ery*^tlr9–/–^* mice developed hypothermia (Figure 1C, *P* = 0.035). CS injection led to mortality in WT and Ery*^tlr9–/–^* mice (Figure 1D). Bacterial dissemination was measured by quantifying CFU in distant organs following CS injection. We observed increased bacterial dissemination in the spleens of Ery*^tlr9–/–^* mice compared with WT mice (*P* = 0.03, Table 1).

We next examined the organs of mice subjected to the sepsis model, 24 hours after CS injection. Consistent with previous reports, splenic injury was characterized by neutrophilic inflammation, lymphocyte death, and germinal hyperplasia (Supplemental Figure 2, A and B and Supplemental Table 1). Liver injury, characterized by hepatocyte apoptosis, oval cell hyperplasia, and focal necrosis, was also present (Supplemental Figure 2, C and D and Supplemental Table 1). Notably, liver microabcesses were present in 2 of the Ery*^tlr9–/–^* mice but none of the WT mice.

We next asked whether RBCs bound live bacteria following CS injection. RBCs were not associated with live bacteria, as we could not culture bacteria from purified RBCs obtained from control or CS-injected mice (Supplemental Figure 2E). Because we had previously observed RBC acquisition of mtDNA during human and murine sepsis (1), we asked whether RBCs acquired bDNA in a murine model of polymicrobial sepsis by performing quantitative PCR (qPCR) for 16S on RBCs obtained from mice subjected to the CS model of sepsis. Consistent with our observation of increased mitochondrial DNA (mtDNA) acquisition during sepsis, 16S was elevated on RBCs from WT mice but not on those from Ery*^tlr9–/–^* mice 6 hours following the induction of CS-induced sepsis. RBCs from the Ery*^tlr9–/–^* mice did not acquire additional microbial DNA after CS injection, suggesting that RBCs may have acquired microbial DNA during sepsis through Tlr9 (Figure 1E). While RBC-bound 16S significantly differed between WT and Ery*^tlr9–/–^* mice following CS-induced sepsis, plasma 16S was not significantly different between WT and Ery*^tlr9–/–^* mice (Supplemental Figure 2F).

Since we previously observed morphologic changes in human RBCs following excess DNA binding (1) and we detected elevated microbial DNA bound to RBCs during sepsis, we asked if RBC morphology is altered following CS-induced sepsis. Notably, Ery*^tlr9–/–^* RBCs remained morphologically intact, whereas echinocytes and loss of biconcave shape were observed in WT RBCs (Figure 1, F and G). These findings indicate that RBC morphological changes observed during sepsis were influenced by RBC Tlr9. We also measured cell-free hemoglobin, which has been shown to increase tissue injury following polymicrobial sepsis (7). We found that plasma cell-free hemoglobin was elevated in Ery*^tlr9–/–^* but not WT mice following CS-induced sepsis (Figure 1H).

### Host cytokine responses are heterogenous and dependent on RBC Tlr9.

**Figure 2:**
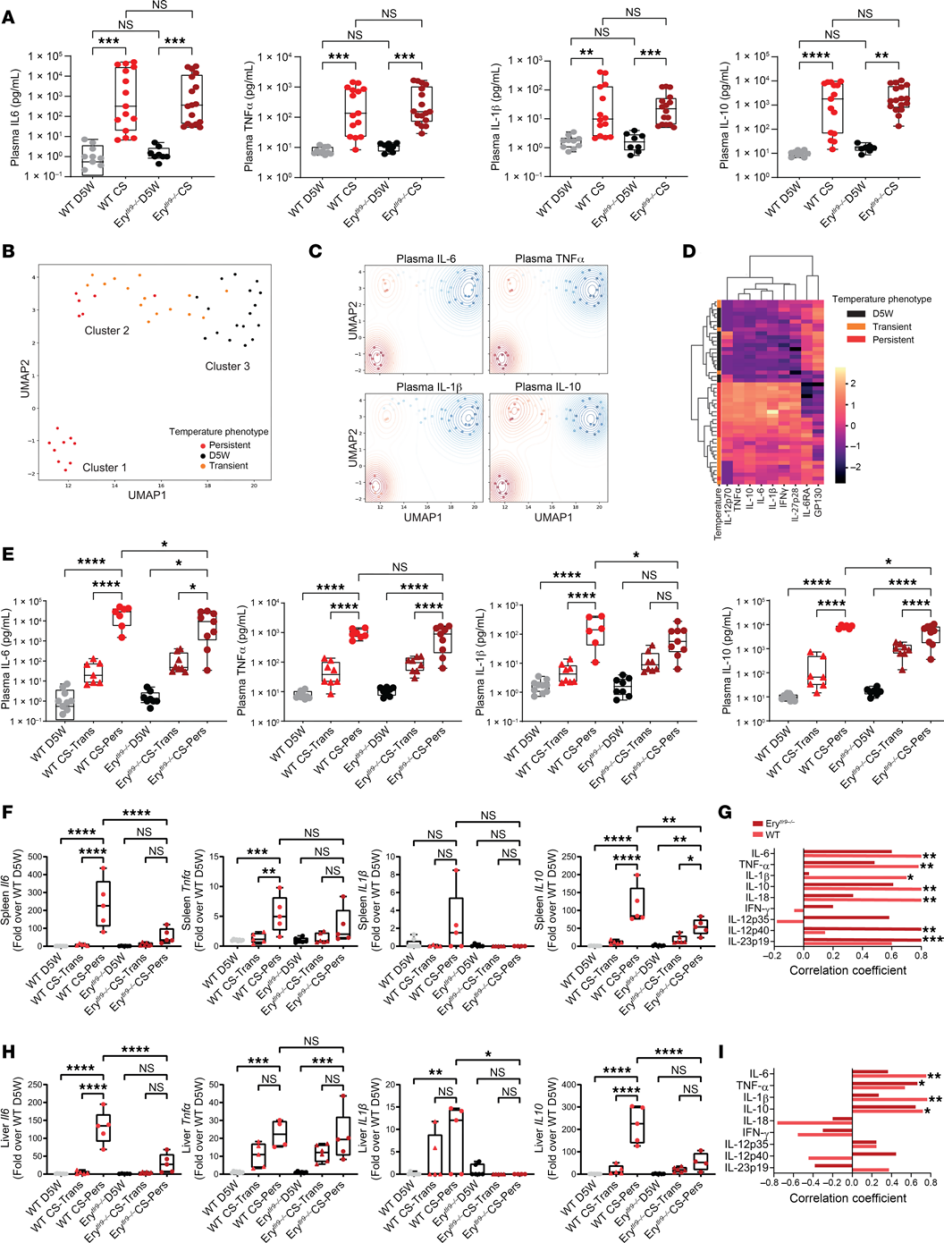
Plasma cytokine levels and gene expression in WT and Ery*^tlr9–/–^* mice following CS injection. (**A**) Plasma cytokine levels 24 hours after CS or D5W injection. *n* = 8–17 per group from 4 independent studies. (**B**) UMAP derived from the 9 measured plasma cytokine concentrations colored according to whether the mice developed persistent or transient hypothermia or were controls (D5W). (**C**) Weighted kernel density estimation illustrating relative concentrations (red higher, blue lower) of the cytokines in each cluster. (**D**) Heatmap of cytokines structured with agglomerative clustering similarly illustrating the correlation between cytokine concentration and hypothermia. (**E**) Plasma cytokine levels 24 hours after CS or D5W injection; plasma cytokines were stratified on the basis of hypothermic state. Trans, transient hypothermia; Pers, persistent hypothermia 24 hours after injection. (**F**) Splenic expression of immune genes stratified according to the hypothermic state. (**G**) Association between bacterial load and tissue cytokine levels in the spleen. Spearman’s correlation coefficient was determined, with an asterisk denoting a significant correlation between tissue cytokine levels and bacterial load. (**H**) Hepatic expression of immune genes stratified on the basis of hypothermic state. (**I**) Association between bacterial load and tissue cytokine levels in the liver. Spearman’s correlation coefficient was determined, with an asterisk denoting a significant correlation between tissue cytokine levels and bacterial load. *n* = 4–8 per temperature-stratified group from 4 independent studies (**F**–**I**). *P < 0.05, **P < 0.01, ***P < 0.001, and ****P < 0.0001, by 1-way Kruskal-Wallis test with Dunn’s post hoc test (**A**) and 1-way ANOVA with Holm-Šidák test (**E**, **F**, and **H**).

Next, we measured plasma cytokine levels following CS injection. We observed heterogeneity in plasma cytokine production in the CS-injected mice (Figure 2A). To better dissect the heterogeneity observed, we applied uniform manifold approximation and projection (UMAP) in order to visualize the 9 measured cytokines in a 2D space. Three distinct clusters emerged in this projection, which discriminated between persistent temperature phenotype and controls (Figure 2B). Cluster 1 was defined by persistent hypothermia and associated with increased organ dissemination and elevated plasma cytokines (Figure 2B and Supplemental Figure 3). Cluster 2 was defined by transient hypothermia and was associated with decreased organ dissemination and decreased cytokine production (Figure 2B and Supplemental Figure 3). Temperature trajectories for individual mice in the different treatment groups shown in Figure 1B are replotted in Supplemental Figure 3A. To visualize the relative concentration of the cytokines in each cluster, we fit a kernel density estimator to the UMAP space weighted by each of the log-normalized features, which revealed cluster 1 to be enriched with the proinflammatory cytokines (Figure 2C). Agglomerative clustering on the 9 cytokines recovered the same clusters (Figure 2D), demonstrating that there were 2 distinct cytokine expression patterns in response to the same insult and that these patterns correlated well with the temperature response phenotype.

Having identified this temperature response cluster, we reclassified mice on this clinically identifiable trait and compared the cytokine response by strain within each group. Analysis based on the hypothermic cluster revealed aberrant cytokine production in the persistently hypothermic Ery*^tlr9–/–^* mice when compared with persistently hypothermic WT mice. We observed attenuated plasma IL-6, IL-10, and IL-1β levels in Ery*^tlr9–/–^* mice compared with WT mice (Figure 2E). We measured cytokine transcript levels in spleen and liver by qPCR. In the spleen, we observed attenuated IL-6 and IL-10 expression, whereas in the liver, we observed attenuated IL-6, IL-1β, and IL-10 production (Figure 2, F–I). Splenic IL-12 family cytokine transcripts levels were also attenuated in Ery*^tlr9–/–^* mice (Supplemental Figure 3C). Because the erythropoietin receptor is present on some nonerythroid cell populations including red pulp macrophages (RPMs) and we observed striking differences in dissemination and cytokine production in spleens between WT and Ery*^tlr9–/–^* mice, we analyzed RPMs from WT and Ery*^tlr9–/–^* mice to ascertain whether *tlr9* was deleted in RPMs (8). As seen in Supplemental Figure 1G, RPMs in Ery*^tlr9–/–^* mice did not show reduced *tlr9* expression. Thus, the differences in dissemination and tissue cytokine production appeared to be a result of RBC Tlr9 deletion.

We examined correlations between tissue and plasma cytokines to determine whether plasma cytokines reflect tissue cytokine production. Plasma IL-6, TNF-α, and IL-10 levels correlated with spleen and liver cytokine generation (Supplemental Table 2). However, spleen and liver IL-1β levels were concordant with plasma levels in WT but not Ery^tlr9–/–^ mice, and tissue IFN-γ levels were discordant with plasma levels in both WT and erythroid Tlr9–deficient mice (Supplemental Table 2), suggesting that plasma IFN-γ may not be a surrogate for tissue-level pathology during sepsis. Because we observed differences in organ dissemination between WT and Ery*^tlr9–/–^* mice, we next asked whether tissue cytokines correlated with bacterial dissemination in remote organs in WT and Ery*^tlr9^^–/–^* mice. We observed significant correlations between tissue bacterial dissemination and cytokines in the spleen and liver of WT and Ery*^tlr9–/–^* mice. However, the cytokine and bacterial dissemination correlations differed between the 2 groups (Figure 2, G and I), with significant associations between tissue IL-6 and IL-1β cytokine production and dissemination observed in WT but not Ery^tlr9–/–^ mice. Collectively, these findings suggest that RBC Tlr9–mediated DNA delivery may have contributed to the heterogeneous host responses to identical stimuli.

### RBCs deliver DNA to immune cells, initiating inflammatory responses.

**Figure 3:**
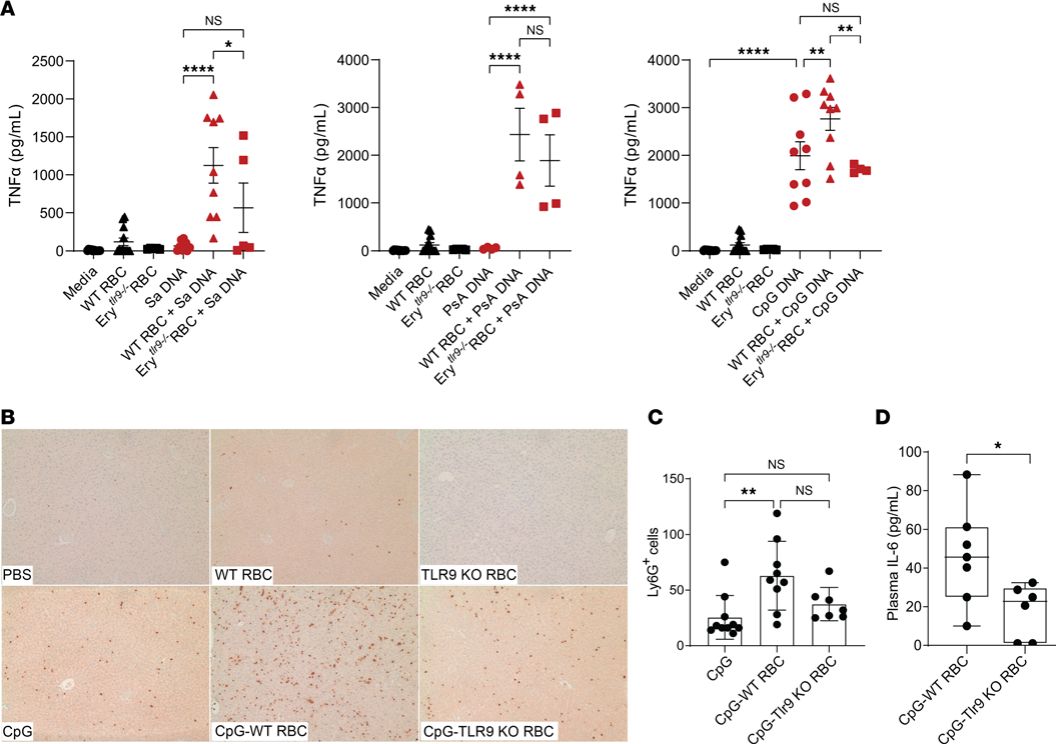
Murine RBCs acquire and deliver DNA to remote organs and immune cells. (**A**) TNF-α production by peritoneal macrophages 4 hours following treatment with media, RBCs (WT or Ery*^tlr9–/–^*), or RBCs preincubated with *S. aureus* genomic DNA (Sa DNA), *P*. *aeruginosa* DNA (PsA), or CpG–ODN 1826 (CoG DBA). Results from 2–4 independent studies are shown. *P < 0.05, **P < 0.01, and ****P < 0.0001, by 1-way ANOVA followed by Holm-Šidák post hoc comparison. (**B**) Ly6G staining of liver sections 6 hours following transfusion of CpG or CpG-treated RBCs. Original magnification, ×10. (**C**) Quantification of Ly6G^+^ cells. (**D**) Plasma IL-6 levels 6 hours after transfusion of mice with CpG-treated WT or TLR9-KO RBCs. *P < 0.05 and **P < 0.01, by Kruskal-Wallis test and Dunn’s post hoc analysis (**B**) and unpaired, 2-tailed *t* test (**D**). *n* = 6–10 from 2 independent experiments.

To validate our observations in the sepsis model, we asked whether RBCs could increase the delivery of microbial DNA to immune cells. We first assessed the ability of RBCs to sequester pathogen-derived DNA; RBCs were incubated with known quantities of bDNA and then assayed for bDNA acquisition by qPCR. In vitro, we found that murine RBCs bound genomic DNA from common bacterial pathogens (Supplemental Figure 4A). We next treated peritoneal macrophages with genomic DNA from *Staphylococcus aureus*, *Pseudomonas aeruginosa*, or the immunostimulatory ODN CpG 1826 or DNA-treated WT or Tlr9-deficient RBCs. Microbial DNA alone did not result in macrophage activation as measured by TNF-α secretion. While microbial DNA alone did not lead to TNF-α release, *S. aureus* DNA carrying WT RBCs, but not *S. aureus* DNA–treated, Tlr9-deficient RBCs, induced robust TNF-α secretion (Figure 3A). Likewise, *P. aeruginosa* DNA alone did not result in macrophage activation or TNF-α release. However, immunostimulatory CpG did result in robust macrophage activation. WT RBCs, but not Ery*^tlr9–/–^* RBCs, augmented CpG-mediated macrophage activation. To verify that our findings were not due to the inflammatory effects of heme, we measured cell-free hemoglobin in the supernatant of RBC-treated macrophages. We did not observe increased hemolysis in the microbial DNA–treated RBC group (Supplemental Figure 4B).

We previously found that CpG-treated RBCs can trigger innate inflammatory responses in naive mice (1). We now asked whether RBC Tlr9 mediated DNA delivery to remote organs in naive mice. Administration of WT RBCs and Tlr9-KO RBCs alone did not lead to liver inflammation as measured by increased neutrophil recruitment, while CpG did lead to increased liver neutrophil recruitment. CpG-treated WT RBCs increased liver neutrophils when compared with RBCs or CpG alone, whereas CpG-treated Tlr9-KO RBCs did not increase liver neutrophil recruitment (Figure 3, B and C). These data would suggest that RBCs through Tlr9 can deliver immunostimulatory DNA to remote organs, triggering inflammatory cell recruitment. Consistent with these results, infusion of CpG-treated Tlr9-KO RBCs resulted in decreased plasma IL-6 production when compared with infusion of CpG-treated WT RBCs (Figure 3D). Collectively, these findings suggested that RBCs are capable of supplying microbial DNA to immune cells and inciting inflammatory cytokine production.

### RBC-bound microbial DNA community richness but not DNA burden correlates with IL-6 in human sepsis.

**Figure 4:**
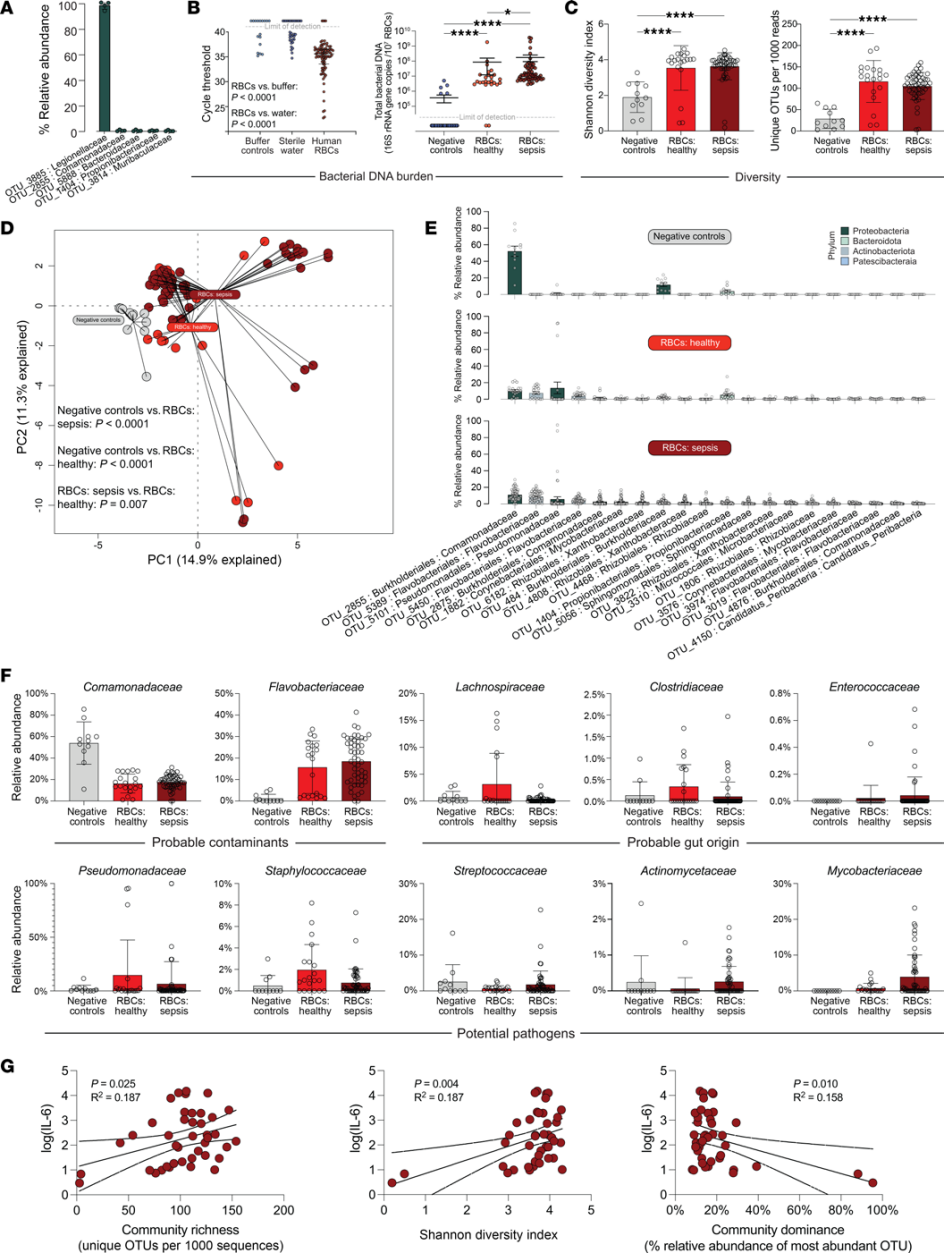
Analysis of bDNA associated with RBCs. (**A**) RBCs were incubated with *Legionella* sp. followed by 16S rRNA gene amplicon sequencing on the RBCs. RBC-associated DNA was dominated by *Legionella*-classified amplicons (97.5%). (**B**) RBC-associated bDNA was quantified by qPCR of the 16S rRNA gene. Human RBCs had a greater quantity of bDNA than did negative control specimens, and RBCs from patients with sepsis had more bDNA than did RBCs from healthy volunteers. *n* = 27 healthy donors and *n* = 64 patients with sepsis. (**C**) RBC-associated bDNA contained a greater diversity of bacterial taxa than did negative control specimens. Negative control specimens included ddH_2_O, AE buffer, AE buffer run through DNA isolation columns, and DNA-free water. (**D**) Bacterial taxa detected in RBCs (both in health and sepsis) were distinct from those of negative control specimens and distinct from each other. (**E**) Abundance rank analysis demonstrated the influence of some contaminant taxa on RBC taxa (e.g., *Comamonadaceae*) as well as distinct taxa within RBC specimens not detected in negative control specimens. (**F**) Direct comparison of prominent bacterial families across negative controls and RBC from healthy individuals and patients with sepsis. (**G**) Among patients with sepsis, the acute inflammatory cytokine IL-6 was positively correlated with RBC-bound bDNA diversity. Unadjusted association of plasma IL-6 with community richness, the Shannon index, and community dominance are shown. When adjusted for the Acute Physiology and Chronic Health Evaluation (APACHE) score and vasopressor use, the association remained significant. Adjusted for the APACHE score: *P* = 0.039, *P* = 0.012, and *P* = 0.024 for richness, the Shannon index, and community dominance, respectively. Adjusted for vasopressor use: *P* = 0.04, *P* = 0.006, and *P* = 0.013 for richness, the Shannon index, and community dominance, respectively. *n* = 20 healthy controls; *n* = 51 patients with sepsis (**C**–**G**).

To determine whether our preclinical observations of RBC-mediated immunoregulation through DNA binding and delivery are germane to human sepsis, we asked whether human RBCs are associated with microbial DNA in health and in sepsis (Figure 4). First, we incubated human RBCs with *Legionella* sp. DNA in vitro to confirm that human RBCs are capable of binding bDNA. When we assayed these RBCs using 16S rRNA gene amplicon sequencing, 97.5% (SD 1.5%) of all RBC-associated sequences were classified as *Legionella* sp. (Figure 4A). We thus concluded that human RBCs are capable of binding bDNA and being assayed via 16S rRNA gene amplicon sequence.

We next investigated the quantity and diversity of bDNA associated with human RBCs using qPCR of the bacterial 16S gene. As shown in Figure 4B, human RBCs contained more detectable bDNA than did our negative controls (buffer controls, *P* < 0.0001; sterile water, *P* < 0.0001). When we compared the quantity of bDNA detected on RBCs from patients with and without sepsis, we found a greater quantity of bDNA on RBCs from patients with sepsis (Figure 4B). Baseline characteristics of the sepsis cohort are found in Table 2. Next, using 16S rRNA gene amplicon sequencing, we compared the community diversity of bDNA associated with human RBCs (metadata can be found in Supplemental Tables 4–12). As shown in Figure 4C, community diversity (both as measured using the Shannon diversity index and by community richness) was greater in human RBC–associated bDNA than in negative control specimens. Neither diversity index differed across humans with and without sepsis. We thus concluded that human RBCs contained more bDNA and greater bacterial diversity than did background control specimens and that the quantity of RBC-associated bDNA was greater in sepsis than in health.

We then assessed the community composition of bDNA detected in association with human RBCs (Figure 4, D–F). Whether visualized via principal components analysis (Figure 4D) or rank abundance (Figure 4E) or tested using permutational multivariate ANOVA (PERMANOVA), we found distinct bacterial communities when comparing negative controls and human RBCs, in both health and sepsis (*P* < 0.0001 for both, PERMANOVA). Communities detected in RBCs from healthy individuals differed from those detected in RBCs from patients with sepsis (*P* = 0.007, PERMANOVA). As shown via rank abundance analysis (Figure 4E), some contaminant taxa detected in negative controls (e.g., *Comamonadaceae*) were detected in RBCs from healthy patients and patients with sepsis, although at a lower relative abundance (>50% in negative controls vs. 10%–15% in RBC specimens). In contrast, numerous taxa were detected in RBC-associated communities that were minimally present in negative controls (e.g., *Pseudomonadaceae*, *Mycobacteriaceae*). We thus concluded that, while as a low-biomass specimen bDNA associated with human RBCs is vulnerable to sequencing contamination, it also has a distinct bacterial signature that cannot be wholly explained via contamination.

Next, we determined the source of this distinct bacterial signal in RBC-associated bDNA (Figure 4F). We directly compared prominent bacterial families detected in RBCs, as compared with negative control specimens, grouped by probable source community. Much of the bacterial signal was suspicious for procedural or sequencing contamination, as evidenced by the high relative abundance of *Comamonadaceae* spp. (detected in negative controls) and *Flavobacteriaceae* spp. (minimally present in negative controls but taxonomically suggestive of an occult source of contamination). However, we found some bacterial taxa enriched in RBC-associated DNA suggestive of probable gut origin (e.g., *Lachnospiraceae*, *Clostridiaceae*, and *Enterococcaceae* spp.). Finally, some bacterial taxa were classified as potential pathogens (e.g., *Pseudomonadaceae* spp., *Staphylococcaceae* spp., *Streptococcacaeae* spp., *Actinomycetaceae* spp., and *Mycobacteriaceae* spp.). Of these, *Streptococcacaeae* spp., *Actinomycetaceae* spp., and *Mycobacteriaceae* spp. were most enriched in RBCs from patients with sepsis relative to those from healthy individuals. Importantly, among patients with sepsis, neither the diversity, quantity, or community composition of RBC-associated bDNA differed across patients with or without culture-identified bacteremia (Supplemental Figure 5, *P >* 0.05 for all comparisons).

We next compared RBC-associated microbiota with patients’ plasma concentrations of IL-6. We found that the community richness of RBC-associated bDNA (the number of unique bacterial taxa detected) was positively correlated with patients’ IL-6 concentrations, explaining the12% variation among patients for this inflammatory cytokine (Figure 4G). Using PERMANOVA, we found that the community composition of RBC-associated bacteria was correlated with patients’ IL-6 concentrations, both at the operational taxonomic unit (OTU) and family level of taxonomic classifications (*P* = 0.03 and 0.01, respectively). The association between RBC-associated bDNA diversity and plasma IL-6 remained significant after adjusting for vasopressor use and severity of illness (Figure 4G).

Taken together, we concluded that human RBCs are capable of binding bDNA and contain a greater quantity and diversity of bDNA than do negative control specimens. The identity of RBC-associated bDNA is influenced — but not entirely explained — by procedural and sequencing contamination, and the identity of bDNA in RBCs from patients with sepsis differs from that of healthy individuals and is correlated with systemic IL-6 concentrations.

## Discussion

In this study, we identify RBCs as critical regulators of the host inflammatory response during sepsis. Using a preclinical model of sepsis and genetic deletion of erythrocyte *Tlr9*, we demonstrate that circulating red cell–mediated DNA delivery drives heterogeneous host inflammatory responses through DNA capture and delivery to remote organs. In vitro, RBCs bound microbial DNA and increased DNA delivery to phagocytes, triggering inflammation. Moreover, RBCs from patients with sepsis had distinct microbial DNA profiles, and we identified RBCs as a distinct reservoir for microbial DNA. These data demonstrate what we believe to be a previously unknown role for circulating RBCs as couriers of microbial DNA, capable of inciting heterogeneous host inflammatory responses during sepsis.

There is renewed interest in antiinflammatory treatments for sepsis following the recent success of antiinflammatory treatments for COVID-associated sepsis and acute respiratory distress syndrome (ARDS) (9–11). Clinical trials of anti–IL-6 and reanalysis of IL-1RA for sepsis have yielded insights into the importance of these pathways in the host inflammatory response to pathogens in specific subsets of patients (12). However, large trials of antiinflammatory agents for sepsis have yet to show a mortality benefit. Discussions surrounding the failure to translate preclinical trials to successful therapies for sepsis have centered around the heterogeneous nature of this clinical syndrome and the inability of animal models to fully demonstrate the clinical features of this complex syndrome. Thus, animal modeling of clinical syndromes such as sepsis has been intensely scrutinized (13). Identifying novel mechanisms that trigger inflammation within sepsis patients offers the promise of precision medicine–based approaches for this complex syndrome. Here, using a preclinical model of sepsis, erythroid TLR9–deficient mice, in vitro studies, and studies in humans, we demonstrate that animal models can recapitulate key clinical features of sepsis and even provide insight into heterogeneous host responses. There was heterogeneity in the inflammatory response to CS injection in WT and in Ery*^tlr9–/–^* mice. However, further studies will be required to elucidate additional mechanisms contributing to the heterogenous response to an identical stimulus. Notably, distinct differences in tissue and systemic inflammatory cytokine production were observed in the absence of RBC TLR9 in the persistently hypothermic cluster. This observation implies that the mechanism of RBC-mediated DNA delivery plays a significant role in eliciting part of the host’s inflammatory response in this severe cluster. These observations were only possible after our unbiased analysis of plasma cytokines demonstrated differential clustering based on the persistent hypothermic response leading to a reanalysis of subjects in this temperature group. These findings are consistent with recent reports suggesting that temperature trajectories predict clinical outcomes (4, 14). Together, these results underscore the critical role of preclinical models in identifying mechanistic elements (i.e., RBC TLR9) that may drive differential host inflammatory responses and identify RBC TLR9–mediated DNA regulation as a potentially treatable trait in sepsis.

In the polymicrobial sepsis model, we observed a marked reduction in cytokine production in the plasma and spleen, alongside increased organ dissemination in mice that lacked erythrocyte Tlr9. Interestingly, plasma cell-free hemoglobin was elevated in the Ery*^tlr9–/–^* mice, which may have contributed to the increased bacterial dissemination observed in this group. We recently demonstrated that human RBCs acquiring CpG DNA become resistant to osmotic lysis (1). In this in vivo model of polymicrobial sepsis, we now show that WT, but not Ery*^tlr9–/–^*, mice acquired microbial DNA and underwent echinocytosis. Whether microbial DNA acquisition and subsequent echinocytosis by RBCs confers resistance to hemolysis remains an active area of investigation.

In vitro experiments showed that WT RBCs could activate macrophages in the presence of bDNA, whereas RBCs from erythrocyte-specific Tlr9-KO (Ery^tlr9–/–^) mice did not. These data indicate that Tlr9-mediated DNA delivery by RBCs plays a crucial role in regulating the host’s inflammatory response. Additionally, in a focused model examining DNA delivery by RBCs, the absence of RBC Tlr9 impaired neutrophil recruitment to the liver following systemic DNA administration. Collectively, these findings underscore the importance of early RBC-mediated DNA delivery in driving the host inflammatory response. A failure in this process, as observed in the absence of RBC Tlr9, resulted in attenuated cytokine responses and inflammatory cell recruitment, impairing microbial control.

We observed consistent findings in our clinical cohort in which red cell–associated microbial DNA community richness correlated with IL-6, suggesting that RBC-mediated DNA delivery was a driver of the IL-6 response at the tissue level. In addition, we have previously demonstrated that critically ill patients with sepsis demonstrate elevated surface RBC TLR9 and diversity in surface RBC TLR9 expression (1). These findings led us to speculate that RBCs contribute to the host inflammatory response and IL-6 signaling and may contribute to host diversity through intrinsically distinct DNA-binding capabilities. However, further studies of RBC heterogeneity and surface TLR9 expression in larger cohorts will be needed to validate this hypothesis.

Our data show that RBCs can avidly bind microbial DNA from multiple pathogenic organisms in vitro. Furthermore, RBCs acquired microbial DNA in a murine model of polymicrobial sepsis and a cohort of critically ill patients with sepsis. We have previously reported that RBCs sequester mitochondrial DNA during non-COVID and COVID sepsis and that RBC-bound mtDNA is associated with anemia (1, 15). Sequestration of mtDNA was not unexpected, as numerous studies have demonstrated the presence of elevated cell-free mtDNA in the plasma of critically ill patients (16, 17). One study, however, has linked the presence of bDNA in the circulation with outcomes in critically ill patients with COVID-19 (18). The detection of bDNA bound to RBCs in patients with culture-negative sepsis was an unexpected finding but consistent with the literature that demonstrates the presence of cell free (cf) bacterial DNA in the circulation of patients with culture-negative sepsis (19–21). Consistent with these findings, others have found bDNA present in remote organs, including the brain and lung, during sepsis, suggesting that gut microbial translocation contributes to organ injury during sepsis (22–24).

Although we observed increased 16S DNA on RBCs from patients with sepsis when compared with healthy donors, the detection of microbial DNA present on RBCs in the healthy donors suggests that another homeostatic role of RBCs may be to scavenge microbial DNA from the gut or other tissues. Given, the ample evidence for microbial translocation into the circulation during routine tasks such as teeth brushing, exercise, and simply aging (25–28), it is plausible that RBCs may serve to sequester microbial DNA under basal conditions. Currently, the origin of RBC-bound microbial DNA remains enigmatic. Given that RBCs circulate through all tissues, we hypothesize that there are several potential sources of RBC-bound microbial DNA, including direct acquisition from infected tissue sites, uptake of microbial DNA from the circulation during infection, and intermittent transfer of bacteria through common sources of transient bacteremia. Our studies comparing bacterial density, diversity, and community composition in patients with either sepsis or culture-negative sepsis, revealed no significant differences. This finding challenges the assumption that bacteremia is the primary source of RBC-bound microbial DNA. In our cohort, probable pathogens constitute only a small portion of the RBC-enriched taxa. We hypothesize that RBC-bound bDNA may represent bDNA accumulated over the lifespan of the RBCs, as RBC-bound DNA is not dominated by the disseminated pathogens. Our findings suggest that RBCs were not just scavenging pathogen DNA in patients with bacteremia but may instead reflect the cumulative bDNA acquired by the RBCs over their 120-day lifespan. Thus, as opposed to a model in which RBC-associated 16S signal identifies acutely disseminated pathogens, we instead hypothesize that the RBC may serve as a circulating “sponge” that acquires bDNA present in the blood throughout its 120-day lifetime. Although the temporal dynamics of RBC-associated microbial DNA (including degradation and turnover) have yet to be determined, we believe this hypothesis could explain the multiple likely anatomic sources of RBC-associated microbial DNA (Figure 4) and is congruent with our hypothesis that RBCs serve as DNA traffickers via a TLR9-dependent mechanism. Thus, RBC-associated bDNA may instead represent evidence of a regulated, homeostatic process (the host calibrating its systemic immune tone based on the density and identity of intermitted bacteria or bacterial egress from any source) as opposed to a mere artifact of overwhelming infection. This leads us to surmise that the association of bDNA with RBCs may be part of a regulated homeostatic mechanism, wherein the host adjusts its systemic immune response based on the nature of bacteria acquired from these diverse sources. Although speculative at this time, such a mechanism, if confirmed, could redefine our understanding of the interplay between microbial exposure during homeostasis and systemic immune modulation. It is thus not surprising that healthy donors and patients with sepsis exhibited a large amount of nucleic acid on their RBCs. The latter likely reflects the ability of RBCs to sequester DNA from the immediate environment, further supporting the hypothesis that RBCs function to maintain homeostasis by continually scavenging cf-DNA (15). During infection, however, we have recently found that excess CpG binding leads to innate immune activation and inflammation, and others have validated these findings by demonstrating the immunogenicity of CpG-loaded RBCs using in vivo tumor models (1, 29). Our current observations suggest that, during sepsis, in the presence of excess bDNA, RBCs deliver DNA to remote organs, driving inflammation.

RBC-based diagnostics may provide insight into inflammation and injury at the tissue level that is not obtainable from plasma. It is possible that this methodology might lead to novel detection strategies for pathogens using small volume samples, as we detected a high abundance of microbial DNA from just 10^7^ RBCs (~2 μL RBCs). Identification of an easily accessible, low-volume, high-mass template for molecular diagnostics represents what we believe to be a fundamental breakthrough in pathogen diagnostics. However, as it currently stands, 16S rRNA bacterial gene sequencing is not an optimal platform to interpret the microbial signal present on RBCs and translate it into actionable bedside information. This is likely for several reasons. One is that, despite a high bacterial signal on RBCs, finding pathogenic DNA is apparently like finding a needle in the haystack. The amount of DNA might be overwhelmed by the other more predominant bacterial nucleic acids, as described above. Second, there is no guarantee that if pathogenic DNA is sequestered on the RBC surface, it will be the 16S rRNA gene fragment, thus future studies targeting the entire microbial genome to examine RBC-bound microbial DNA will be required to overcome this limitation. Additionally, given the long lifespan of RBCs, it is plausible that microbial DNA associated with the RBCs detected by sequencing reflects cumulative microbial DNA present over the RBC’s lifespan (~120 days). As evidenced by our 16S rRNA gene analysis of human RBCs, this low-biomass specimen is also vulnerable to procedural and sequencing contamination. Hence, in the context of diagnostics, the application of RBC-based 16S sequencing is currently considered to have limited effectiveness. Future research focusing on targeted qPCR for the detection of RBC-bound pathogens may be more beneficial.

Although the quantity of 16S bound to RBCs did not associate with inflammatory cytokines in patients, community composition and richness of RBC-associated microbial DNA did associate with IL-6. Further in vitro mechanistic studies will be needed to interrogate the hypothesis that RBC-bound microbial DNA composition differentially regulates host inflammatory responses including phagocyte cell death. Our clinical observations of RBC-bound DNA associated with inflammation were validated in the preclinical animal model, in which we demonstrated that loss of RBC TLR9 attenuated end-organ IL-6 and IL-1B production in severely ill animals. However, our study has limitations. Although our preclinical data suggest that RBCs can deliver CpG DNA to remote organs, in this single-center, small cohort, we were only able to show an association between RBC-bound microbial DNA diversity and plasma IL-6 levels. Larger studies will be necessary to further elucidate the role of RBC-mediated microbial DNA regulation in influencing host inflammatory responses in patients with sepsis.

In this study, we uncover a non-gas-exchanging function of RBCs as couriers of microbial DNA through TLR9. Our findings show that RBC-mediated DNA transport elicited specific host responses in a preclinical sepsis model and that RBC-bound microbial DNA associated with systemic inflammation in patients with sepsis, offering new insights into previously unexplored mechanisms contributing to the heterogenous host response in sepsis. Additionally, RBCs emerged as an unexpected reservoir for microbial DNA that may be exploited in the future to develop molecular diagnostics for infectious diseases. Collectively, we identify a critical role for circulating RBC TLR9 in modulating host inflammatory responses during sepsis, highlighting their dual function as both reservoirs and couriers of microbial DNA.

## Methods

### Sex as a biological variable.

This study examined male and female mice in the preclinical sepsis model, with similar findings reported for both sexes.

### Sepsis patient cohort.

RBCs were obtained on the day of ICU admission from patients enrolled in the Molecular Epidemiology of Severe Sepsis in the ICU (MESSI) cohort study at the University of Pennsylvania (IRB no. 808542) (1). Patients were eligible if they presented to the medical ICU with strongly suspected or confirmed infection and new or worsening organ dysfunction per historic sepsis-2 “severe sepsis” criteria because enrollment preceded the publication of sepsis-3 (30). Exclusion criteria included the primary reason for ICU admission being unrelated to infection, admission from a long-term acute-care hospital that might select for sepsis survivors, or desire for exclusively palliative measures on ICU admission. Patients or their proxies provided informed consent.

### Experimental animals.

C57BL/6 mice were purchased from the Charles River Laboratories. Tlr9-KO mice were produced by S. Akira and provided by E. Behrens (Children’s Hospital of Philadelphia, Philadelphia, Pennsylvania, USA). Mice lacking erythrocyte Tlr9 (Ery*^tlr9–/–^*) were generated by crossing ErGFPcre mice (a gift from U. Klingmüller, German Cancer Research Center, Heidelberg, Germany) and conditional Tlr9-KO mice (obtained from the European Mutant Mouse Archive). Genotype was confirmed through PCR amplification, and the primers are listed in Supplemental Table 3. All experimental procedures were performed using 8- to 12-week-old mice. Littermate controls were used in all experiments in which Ery*^tlr9–/–^* mice were used.

### Reagents.

Bacterial genomic DNA was obtained from the American Type Culture Collection (ATCC) (25923D-5 for *S*. *aureus* ssp. *aureus* strain Seattle 1945; 47085D-5 for *P*. *aeruginosa* strain PAO1-LAC; 700721D-5 for *K*. *pneumoniae* strain MGH78578). *Legionella pneumophila* was a gift from Sunny Shin (University of Pennsylvania, Philadelphia, Pennsylvania, USA). The corresponding genomic DNA was extracted using a DNeasy kit (Qiagen). Integrated DNA Technologies (IDT) synthesized ODN1826 (CpG).

### Macrophage isolation.

WT mice were injected i.p. with 3 mL Brewer’s thioglycollate media to obtain thioglycollate-elicited macrophages. After 4 days, mice were sacrificed via CO_2_, and peritoneal lavage was aseptically collected by injecting and aspirating 5 mL RPMI-1640 media in the peritoneum twice. The lavage was strained through a 70 μm cell strainer, and the cells were adhered to a tissue culture–treated dish for 1 hour at 37ºC in D10 media (DMEM supplemented with 10% FBS, 1% penicillin/streptomycin, and 1% l-glutamine). Nonadherent cells were removed by washing 3 times with PBS (with calcium/magnesium). Adherent macrophages were lifted with a cell scraper and seeded onto 24-well plates at 250,000 cells per well overnight at 37ºC in D10 media. Macrophages were washed with PBS once before incubation with RBCs. In some experiments, macrophages were seeded onto sterile 12 mm coverslips in 24-well plates.

### In vitro DNA delivery by RBCs.

Freshly isolated, leukoreduced RBCs were incubated with DNA at a ratio of 10 ng bDNA to 10^7^ RBCs in 200 μL DMEM for 4 hours at 37ºC on a nutator. ODN1826 (25 μg/mL) was used as a control. All binding reactions were carried out in Eppendorf DNA LoBind tubes. Subsequently, the DNA-RBC mixture was added to macrophages and incubated at 37ºC for 4 hours. The supernatant containing RBCs were harvested and frozen at –80ºC for ELISA. To evaluate hemolysis, 60 μL of the supernatant was centrifuged at 800*g* for 5 minutes, and 50 μL clarified supernatant was used to quantify cell-free hemoglobin using the QuantiChrome hemoglobin assay according to the manufacturer’s instructions.

### Flow cytometry.

To evaluate the expression of TLR9 in myeloid cells and neutrophils, flow cytometry was performed. Whole blood was collected from WT and Ery*^tlr9–/–^* mice by cardiac puncture and then centrifuged for 10 minutes at 3,000*g*. Plasma was aspirated, and the buffy coat was collected. RBCs in the buffy coat were lysed using ACK lysis solution. Following red cell lysis, 10^6^ cells were washed and then treated with Fc block (BioLegend, catalog 101319) for 30 minutes on ice. Surface staining was performed with either CD11b (BioLegend, clone M1/70, catalog 01251) or Ly6G (BioLegend, clone 1A8, catalog 127626) antibodies for 30 minutes on ice. Cells were washed twice and then fixed and permeabilized using BD Cytofix/Cytoperm solution (BD Biosciences, catalog 554714) for 20 minutes at room temperature. Samples were washed and then intracellularly stained for TLR9 (BioLegend, clone S18025A, catalog 159103) for 40 minutes on ice. Following staining, cells were washed and resuspended in FACS buffer, and flow cytometry was performed using an LSR Fortessa cytometer (BD Biosciences). Analysis was performed using FlowJo Software.

### DNA binding by murine RBCs.

RBCs were isolated as previously described (3). RBCs were incubated with 1 ng bDNA in 200 μL PBS in an Eppendorf DNA lo-bind tube on a nutator at 37ºC for 2 hours. The RBCs were then separated from the supernatant using sucrose-gradient centrifugation (30% sucrose cushion, 13,000*g* for 5 min). The isolated red cell pellets were frozen at –80ºC until DNA extraction with a DNeasy blood kit (Qiagen). After DNA extraction, RBC-associated DNA was quantified with qPCR using QuantStudio 6 or 7 (Applied Biosystems) (the primers and probes are listed in Supplemental Table 3). For *S*. *aureus* or *K*. *pneumoniae*, 16S multiplex primers were used in conjunction with the corresponding species-specific probe (31).

### In vivo DNA delivery by RBCs.

Leukoreduced murine RBCs from WT or Tlr9-KO mice were washed with PBS and concentrated to a hematocrit of 40%. RBCs (160 μL) were mixed with 50 μg ODN1826 at 40 μL and i.v. transfused into recipient mice. After 6 hours, blood was harvested via cardiac puncture, and plasma cytokines were quantified by ELISA. Livers were fixed in formalin and processed for histology as described below.

### CS-induced sepsis.

Ery*^tlr9–/–^* mice and their littermates controls were used. CS was generated as previously described and injected i.p. at 2 mg/kg (1). Body temperature was monitored using an infrared thermometer at specific time points after CS injection (2, 4, 6, and 24 hours), with a detection limit of 88°F. Transient hypothermia in mice was defined as development of a low temperature of 88°F or below at any of the early time points (2, 4, or 6 hours) but recovery to above 90°F by 24 hours. Persistent hypothermia in mice was defined as sustained low temperatures (remaining ≤88°F at 24 hours). At the indicated time point (6 or 24 h after CS or control), mice were euthanized with 80 mg/kg ketamine and 10 mg/kg xylazine. Blood was collected via cardiac puncture with heparinized syringes. Tissues were excised and stored in TRIzol, 10% buffered formalin acetate, or PBS.

Quantification of tissue bacterial load was performed on the same day as necropsy. Tissues were weighed prior to storing in PBS and then homogenized and serially diluted in PBS. Ten microliters of each dilution was plated onto brain-heart infusion agar in triplicate and incubated at 37ºC for 24 hours, and bacterial colonies were counted. Bacterial dissemination was determined to have occurred if greater than 10^5^ CFU/tissue. Isolation of RBCs for bound bacteria quantification were purified as previously described (3) using TER119 beads. To generate RBC smears, 5 μL packed blood was spread onto a SuperFrost Plus microscope slide. The slides were then air-dried and stained using Diff-Quik (Sigma-Aldrich).

### Histology scoring criteria.

Spleens and liver were fixed overnight in formalin. Tissues were then processed for paraffin sectioning and scoring by the Comparative Pathology Core of the University of Pennsylvania School of Veterinary Medicine. Histopathological examination was performed in a blinded manner by a veterinary pathologist. Twenty fields of 400× tissue sections were evaluated for the histological criteria listed in Supplemental Table 1. As no validated sepsis scoring system exists for the liver and spleen, all histological findings were scored in a semiquantitative manner based on severity per International Harmonization of Nomenclature and Diagnostic Criteria (INHAND) recommendations. Criteria considered common findings in sepsis were then weighted higher than less specific lesions and common background changes.

### RBC smear scoring.

The blood smear score was determined by 2 blinded reviewers using an Olympus BX41 microscope at 40× magnification. Five random fields of view were assessed for each mouse. Echinocytes, bite cells, rouleaux, and teardrop cells were considered “abnormal” RBCs. If over 50% of the field of view contained abnormal cells, then that field of view was given a score of 1. If less than 50% of the field-of-view contained abnormal cells, then that field of view was given a score of zero. Once 5 random fields of view were scored for a given mouse, then a total score from 0–5 was determined by summing the individual field-of-view scores for the mouse. A total score of zero indicated low abnormality, whereas a total score of 5 indicated very high abnormality.

### Hemoglobin assays.

Hemoglobin assays were performed using a commercially available kit (QuantiChrom Hemoglobin Assay, BioAssay Systems, catalog DIHB-250). RBCs from naive WT and Ery*^tlr9–/–^* mice were isolated using magnetic anti-Ter119 beads (Miltenyi Biotec). Cells were saved as 10^7^ cell pellets at –80°C until use. To measure hemoglobin in RBCs, 10^7^ cells were lysed in 50 μL water prior to the assay. For measurement of cell-free hemoglobin in the plasma, plasma from animals that received either a 24-hour injection of D5W or CS was saved at –80°C until the time of the assay. Plasma was thawed and then diluted 1:4 in water and assayed following the kit instructions.

### qPCR.

RNA was extracted from tissues in TRIzol (Ambion, Thermo Fisher Scientific) using an RNeasy Plus kit (Qiagen) according to the manufacturer’s protocol. RNA was reverse-transcribed into cDNA using a High-Capacity cDNA kit (Applied Biosystems). DNA on RBC samples was extracted with a DNeasy blood and tissue kit (Qiagen). RBCs were isolated and manually enumerated as previously described (1). Gene expression was quantified with PowerUp SYBR Green Master Mix or TaqMan Fast Universal PCR Master Mix (Applied Biosystems) (primers and probes are listed in Supplemental Table 3). For quantification of 16S rDNA on murine RBCs, a 1:5 dilution of DNA was used for qPCR.

### Cytokine quantification.

Cytokine expression in mouse plasma was quantified with a custom U-plex cytokine panel (Meso Scale Discovery) of the following analytes: IL-6, TNF-α, IL-1β, IL-10, IL-12p70, IL-27p28, and IFN-γ or by standard ELISA according to the manufacturer’s protocol (DuoSet, R&D Systems). If a value was below the limit of detection, it was denoted with a zero.

### Isolation of F4/80^+^ red pulp macrophages.

F4/80^+^ cells were magnetically isolated with F4/80 beads (Miltenyi Biotec) following the manufacturer’s protocol.

### Bioinformatics analysis.

Unsupervised learning was performed to identify clusters within the data. Agglomerative (hierarchical) clustering was applied to the Z-normalized cytokine data with complete linkage to produce heatmaps and dimensional reduction using UMAP. For the latter approach, the Python package “UMAP-learn” was used to calculate the projection using the 9 cytokines as input features. To visualize how the underlying features contributed to the visualized clusters, a kernel density estimator was fitted to the UMAP coordinates weighted by the log of the features’ *z* scores to produce contour maps. Graphics were generated using a combination of the Seaborn and Matplotlib packages.

### 16S qPCR on human RBCs.

RBCs were isolated using magnetic anti–glycophorin A (GPA) beads (Miltenyi Biotec) and then manually enumerated and aliquoted as 10^7^ RBCs and saved at –80°C until extraction. DNA extraction was performed using a DNeasy blood kit (Qiagen), and DNA was eluted in 152 μL buffer AE (10 mM Tris-Cl and 0.5 mM EDTA at a pH of 9.0). 16s DNA was evaluated using the Universal 16s primers (probes are listed in Supplemental Table 3), and PCR was run to 50 cycles and quantified using QuantStudio 6 or 7 (Applied Biosystems). When converting cycle thresholds to copy numbers, undetermined values were reported as “1.” AE buffer and double-distilled water (ddH_2_O) negative controls were run with each qPCR reaction. For 16S sequencing, ddH_2_O, AE buffer, and AE buffer run through DNA isolation columns were included as negative controls. Three individual samples for each negative control were provided to the sequencing core. In addition to these controls, DNA-free water and gene-block negative controls were included in the sequencing.

### 16S sequencing and analysis.

The 16S rRNA amplicon sequences were initially analyzed using QIIME2 (qiime2.org, version 2021.2). DADA2 was used for quality control, denoising, and amplicon sequence variant (ASV) creation (32). ASVd with fewer than 10 hits across samples were filtered out. A naive Bayesian classifier was used to assign taxonomy against the Greengenes (version 13.8) database. Microbial ecology analysis was performed using the vegan package 2.6-1 and *R*, 4.2.2 (33–35). For relative abundance and ordination analysis, samples were normalized to the percentage of total reads, and analysis was restricted to ASVs that were present at greater than 1% of the sample population. All ASVs were included in the diversity analysis. Direct community similarity comparisons were performed using the Bray-Curtis similarity index. Ordinations were performed using principal component analysis on Hellinger-transformed normalized OTU tables generated using Euclidean distances (36).

### Statistics.

GraphPad Prism (GraphPad Software) was used to perform all statistical analyses, with the exception of the 16S sequencing analysis and Bioinformatics analysis described above. A 2-tailed *t* test or Mann-Whitney *U* test was used to compare 2 groups depending on normality. A 1-way ANOVA or Kruskal-Wallis test was used to compare more than 2 groups depending on normality, followed by the post hoc tests indicated in the figure legends. For 16s sequencing analysis the significance of differences in community composition was determined using PERMANOVA (Adonis) with 10,000 permutations using Euclidean distances. The means were compared with a 2-tailed Student’s *t* test and 1-way ANOVA with a Holm-Šidák multiple-comparison test as appropriate. A *P* value of less than 0.05 was considered significant.

### Study approval.

All studies involving animals were approved by the University of Pennsylvania IACUC. All studies involving humans were approved by the University of Pennsylvania IRB. Patients or their proxies provided written informed consent.

### Data availability.

All data are available in the main text and supplemental materials. A Supporting Data Values file can be found online. The metadata can be found in Supplemental Tables 4–12. Raw sequence data have been uploaded to the NCBI’s BioProject database (accession number PRJNA1068992).

## Author contributions

Experiments were conceived and designed by LKML and NSM. Experiments were performed by LKML, KAE, ML, EAO, LS, and JSJ. Data were analyzed by LKML, JSJ, CVC, JM, NJK, NY, NJM, PR, RPD, and NSM. NSM and RPD wrote the manuscript.

## Supplementary Material

Supplemental data

Supplemental tables 4-12

Supporting data values

## Figures and Tables

**Table 1:**
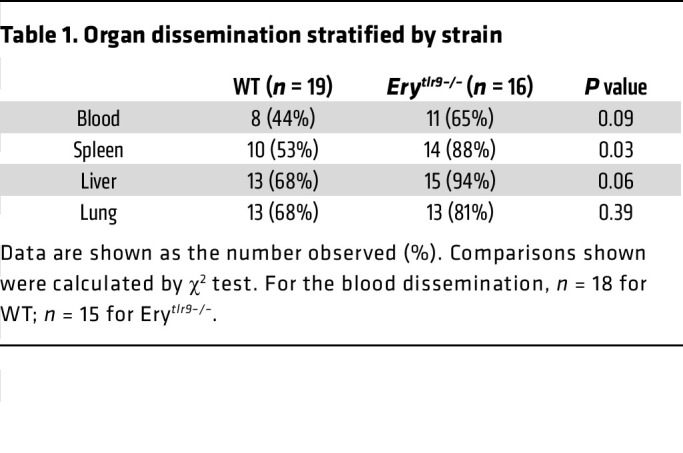
Organ dissemination stratified by strain

**Table 2:**
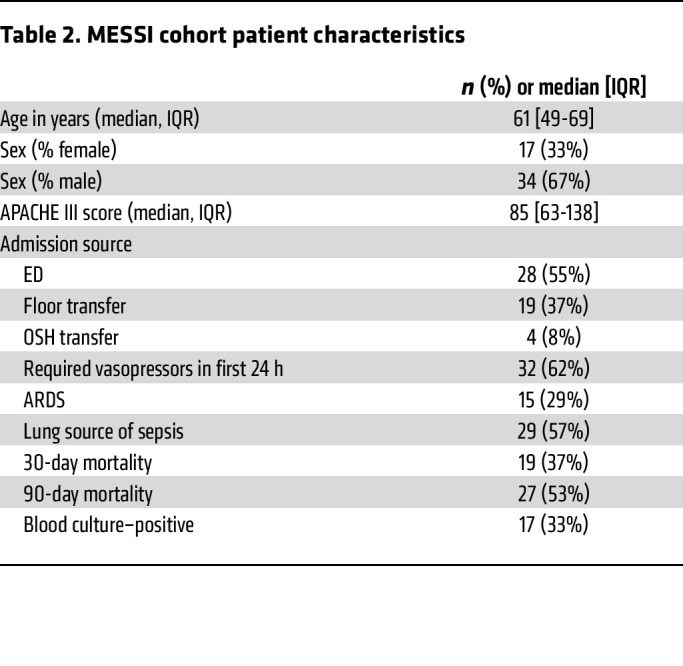
MESSI cohort patient characteristics

## Version 1 12/12/2024

In-Press Preview

## Version 2 02/17/2025

Electronic publication

## Version 3 02/19/2025

Corrected spelling of Dr. John McGinniss
