# Supplementary material for: Red blood cells capture and deliver bacterial DNA to drive host responses during polymicrobial sepsis
Source: J Clin Invest. 2024 Dec 12;135(4):e182127. doi: 10.1172/JCI182127 (PMC11827885; doi:10.1172/JCI182127)
Supplement: Supplemental data [file jci-135-182127-s007.pdf]

Supplemental Material

Supplemental Figure 1

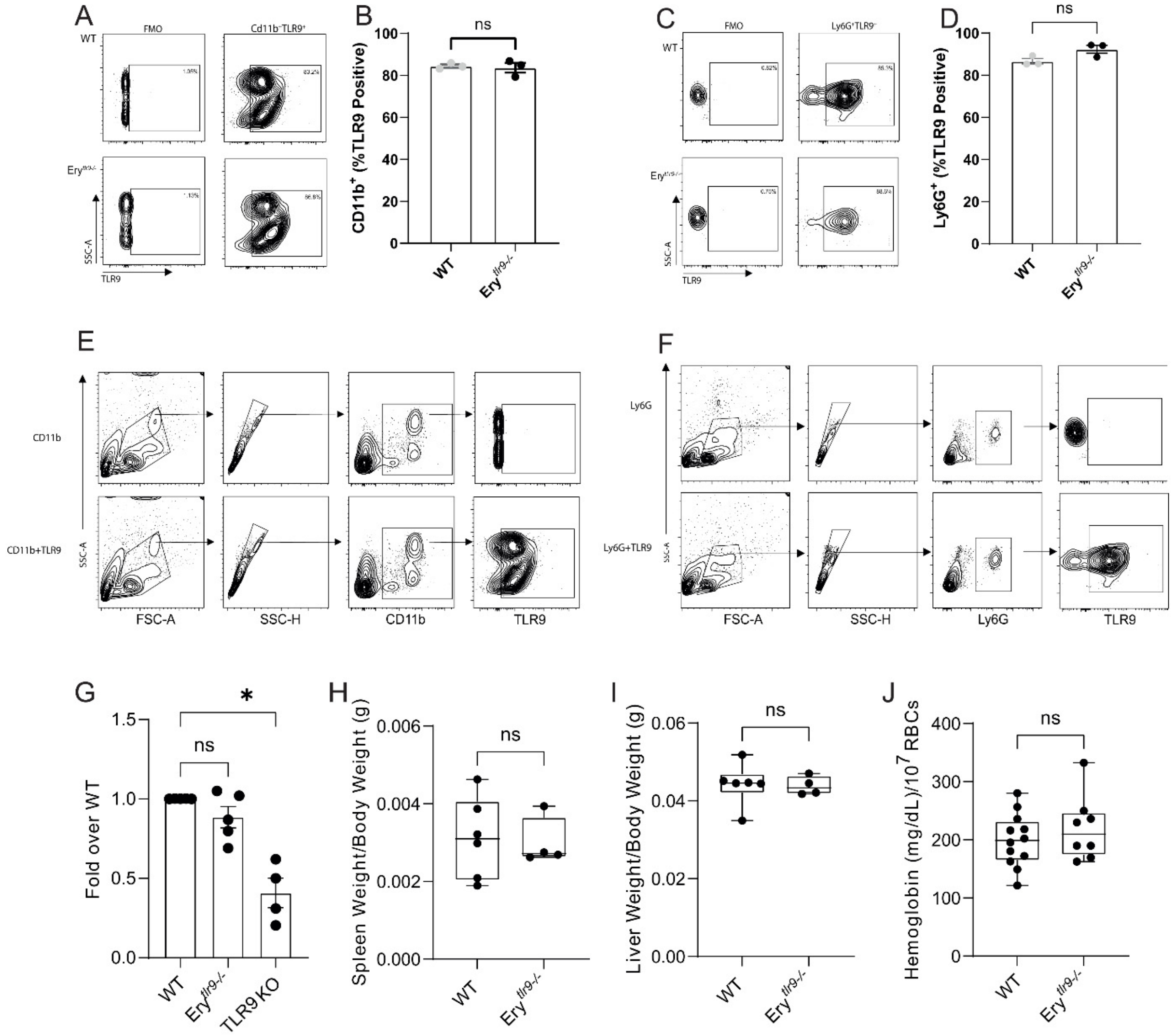

Supplemental Figure 1. TLR9 expression is preserved on immune cells in the Ery$^{tlr9-/-}$ mice and spleen, liver weights, and baseline heme content do not vary between WT and Ery$^{tlr9-/-}$ mice. The buffy coat from from naïve WT and Ery$^{tlr9-/-}$ mice was isolated and RBCs were lysed. Flow cytometry for TLR9 (intracellular staining) was performed. (**A**)TLR9 expression in Cdllb$^+$ cells in a representative WT and Ery$^{tlr9-/-}$ mouse, (**B**) summary statistics of 3 mice/strain. (**C**) TLR9 expression in Ly6G$^+$ cells from a representative WT and Ery$^{tlr9-/-}$ mouse, (**D**) summary statistics of 3 mice/strain. The gating strategy is shown in (**E**) and (**F**). **(G)** F4/80$^+$ cells were purified from the spleen. qRT-PCR analysis of *Tlr9* expression in F4/80$^+$ cells was shown. Differences between groups were analyzed with Kruskal-Wallis test and Dunn's post-hoc analysis, n=4-5 mice, *P<0.05. **(H,I)** WT and Ery$^{tlr9-/-}$ mice liver and spleens were weighed and normalized to total body weight. **(J)** WT and Ery$^{tlr9-/-}$ RBCs were isolated using Ter119 beads and

manually enumerated. RBCs were lysed and free hemoglobin was measured using a cell-free hemoglobin detection kit. Differences between groups were analyzed with paired t-test.

Supplemental Figure 2

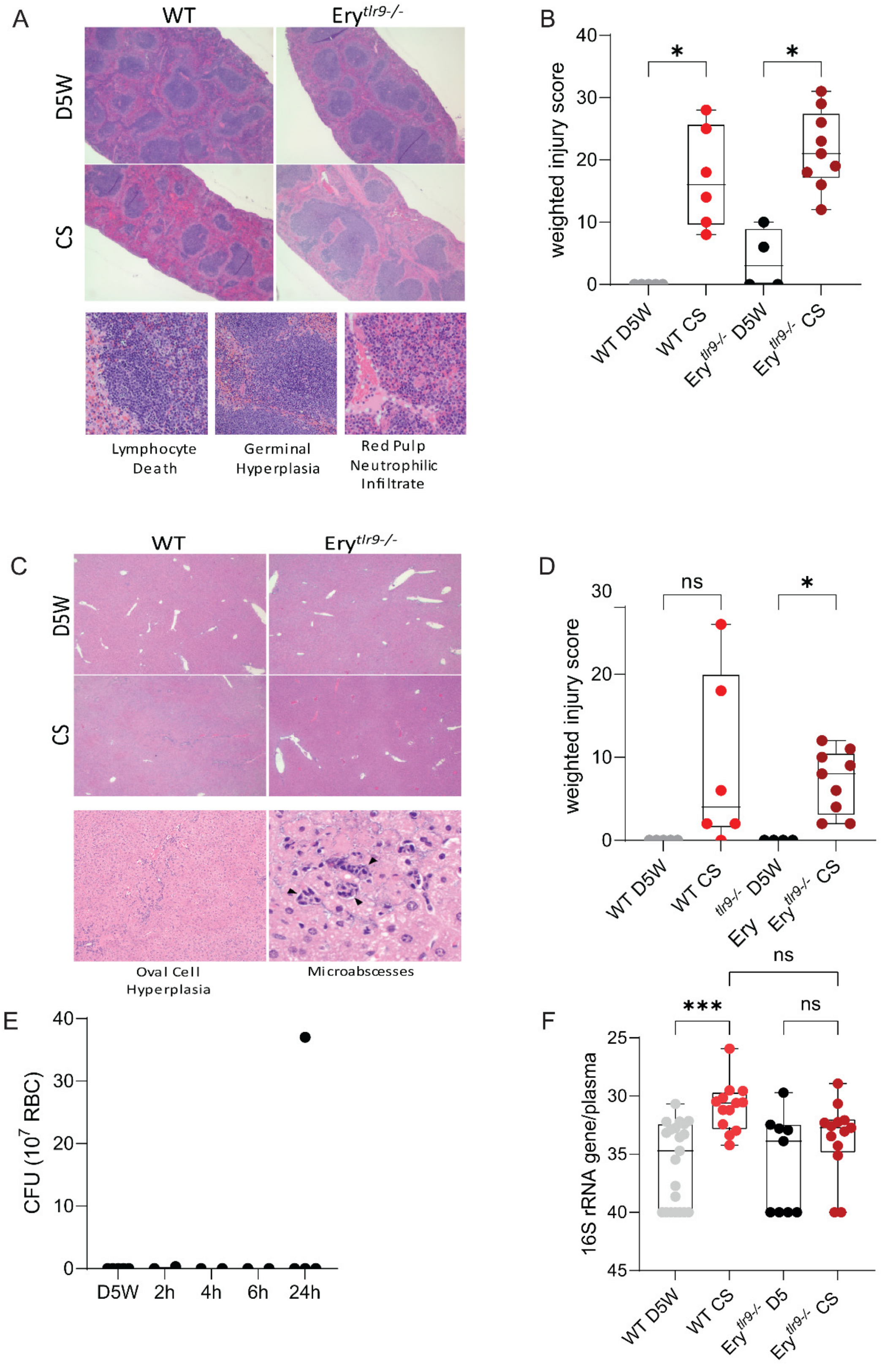

Supplemental Figure 2. Organ injury and plasma microbial DNA following CS-injection of WT and Ery$^{tlr9-/-}$ mice. Organ injury following D5W or CS injection at 24 hours for (**A**, **B**) spleen, (**C**,**D**) liver. Representative H&E sections at 4x objective and high-power micrographs demonstrating specific pathologic features (WT mice shown) are shown in A and C, with corresponding injury scores shown in B and D. Differences between groups were analyzed with the Kruskal-Wallis test and Dunn's post-hoc analysis in (**B** and **D**), n=4-9 from 2 independent studies, *$P<0.05$. **(E)** RBCs do not contain culturable bacteria. RBCs were purified from CS-injected mice using TER119 beads. 10^7 RBCs were plated on BHI agar to quantify RBC-associated bacteria. **(F)** 16S rRNA gene in the plasma is elevated in WT mice 24 ours after CS-induced sepsis but not Ery$^{tlr9-/-}$ mice. Kruskal-Wallis test of all groups, P=0.001, Dunn's multiple comparisons, P=0.0006 for WT D5W v WT CS, all other comparisons were not significant, n=9-21 mice/group, cycle threshold is shown on the graph.

Supplemental Figure 3

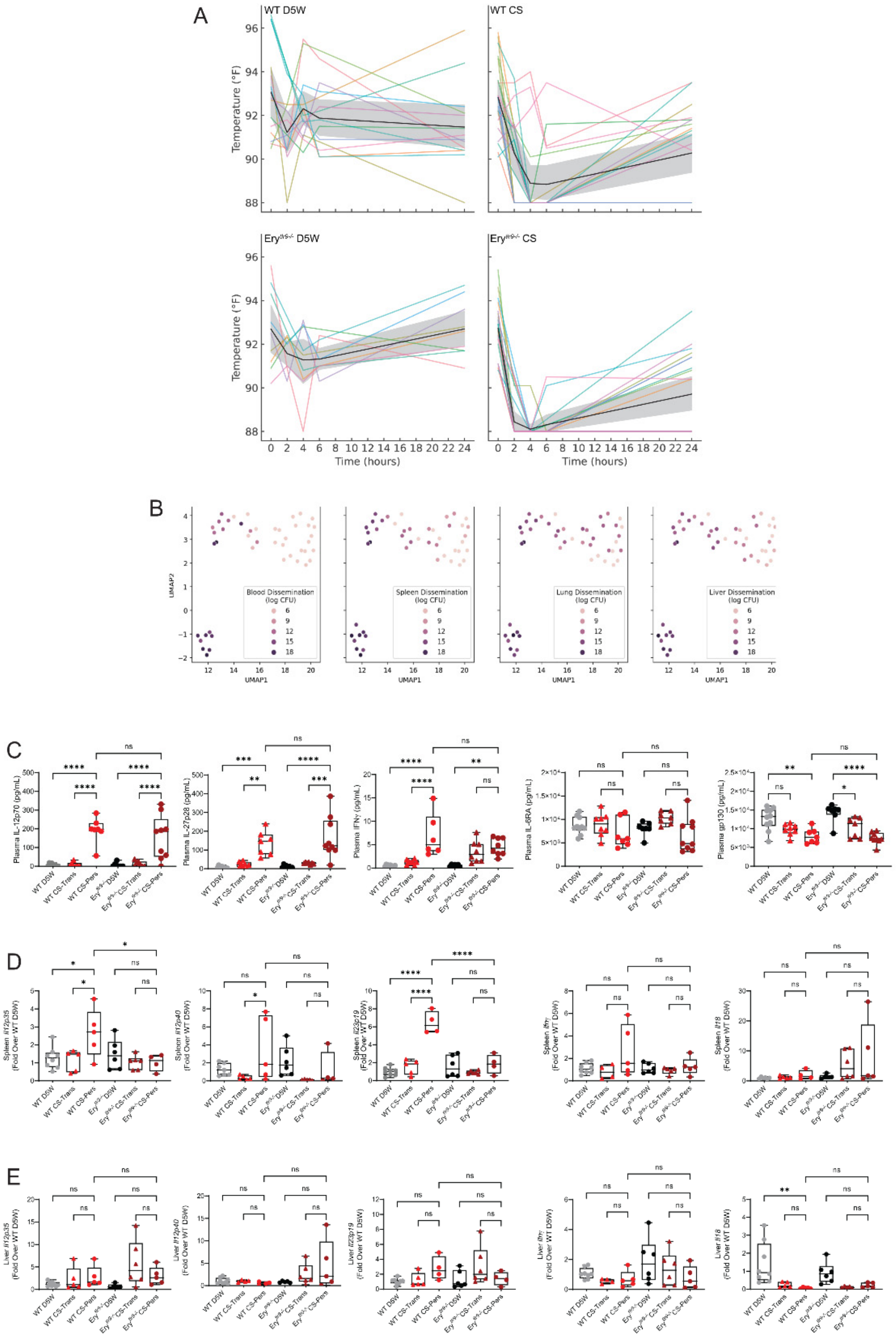

Supplemental Figure 3. Plasma cytokine and tissue gene expression for CS-injected mice. (A) Temperature trajectories for individual mice, the black line represents the standard error of the mean. (**B**) Weighted kernel density estimation illustrating relative bacterial burden in each cluster of mice. (**C**) Plasma cytokine expression for IL-12p70, IL-27p28, IFNγ, IL-6RA, and

gp130 stratified by hypothermic state. Trans=transient hypothermia, Pers=persistant hypothermia at 24 hours following injection. (**D**) Spleen and (**E**) liver expression of IL-12 family cytokines, *Ifng*, and *Il18*, stratified by hypothermic state. Differences between groups were analyzed with the Kruskal-Wallis test followed by Dunn's analysis in (**C**) and one-way ANOVA with holm-Sidak post-hoc test in (**D** and **E**), n=8-17/treatment group from 4 independent studies, P <0.05, **P< 0.01, ***P<0.001, ****P<0.0001.

Supplemental Figure 4

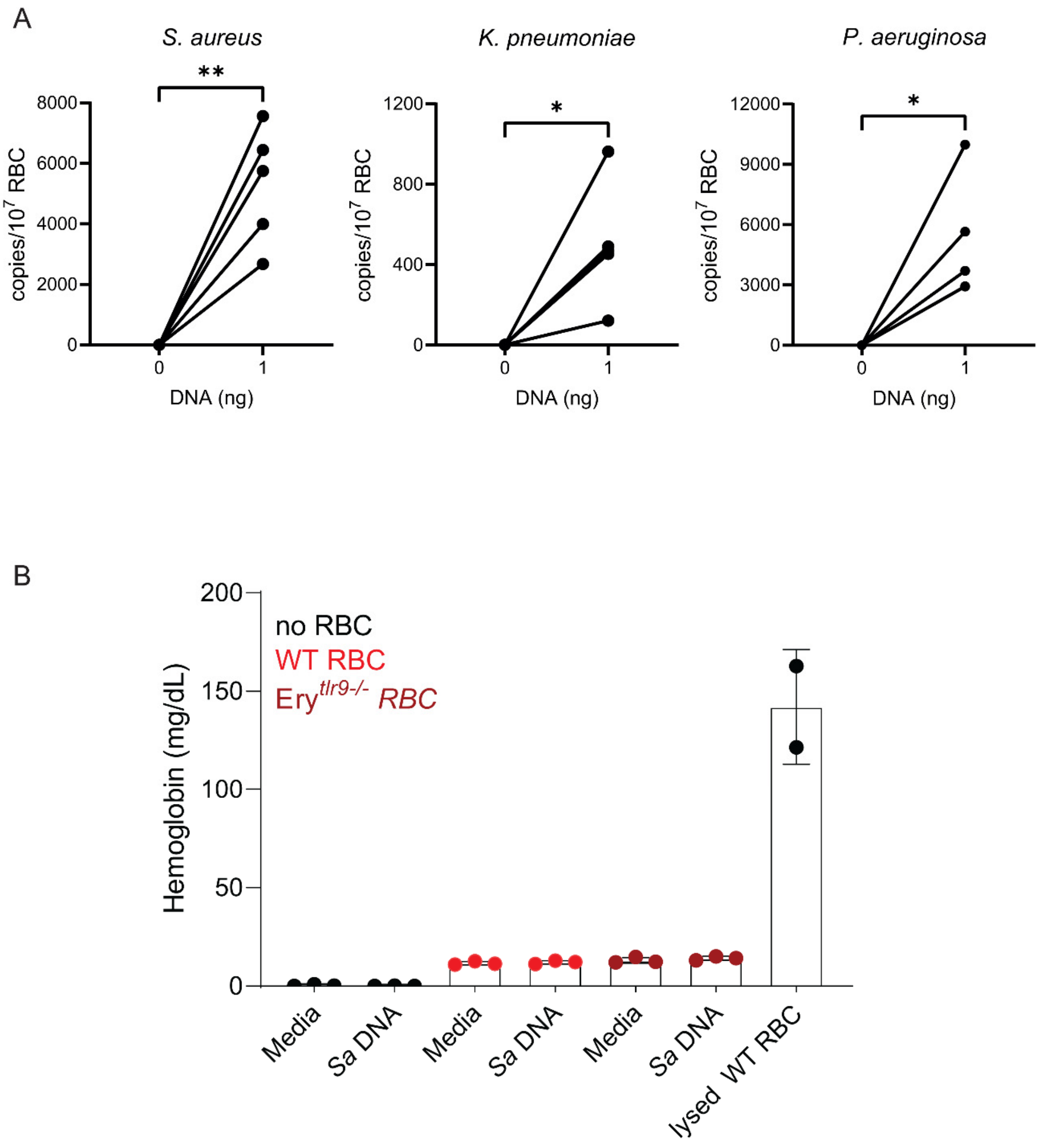

Supplemental Figure 4. RBCs acquire microbial DNA and hemolysis is not increased upon DNA binding and co-incubation with macrophages. (**A**) Acquisition of pathogen derived genomic DNA by murine RBCs (*S. aureus, K. pneumoniae, or P. aeruginosa) in vitro*. Paired- t-test, n=4-5, **P=0.004, *P=0.021,*P=0.019 for *S. aureus, K. pneumoniae, or P. aeruginosa.* (**B**) Cell-free hemoglobin detected in the supernatant of macrophages incubated with media or DNA-treated RBCs. Lysed RBCs are shown as a positive control.

Supplemental Figure 5

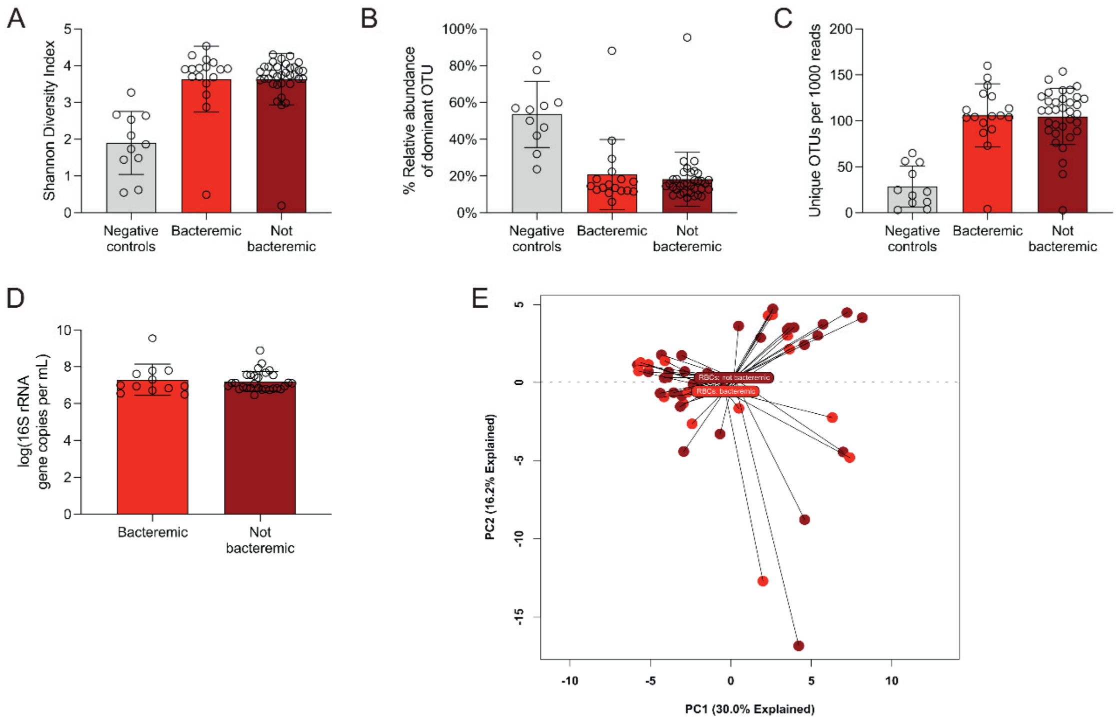

Supplemental Figure 5. Analysis of red cell-associated bacterial DNA in bacteremic and culture-negative sepsis patients. (**A**) diversity of bacterial taxa, (**B**) relative abundance and (**C**) unique OTUs do not differ between bacteremic and culture-negative patients (not bacteremic). (**D**) 16S detected on RBCs. (**E**) Bacterial taxa detected in RBCs from sepsis patients (both bacteremic and not bacteremic) are not significantly different from each other.

Supplemental Table 1. Histologic criteria used to score organ injury

Spleen: Selected Criteria	
Red pulp congestion	Weighted 2x
Red pulp neutrophilic infiltrate	Weighted 2x
Erythrophagocytosis	
Hemosiderophages	
Red pulp fibrin deposition	
Lymphocyte death	Weighted 2x
Mature lymphoid depletion	Weighted 2x
Germinal center hyperplasia	Weighted 2x
Histiocyte infiltration	

Liver: Selected Criteria	
Kupffer cell hyperplasia	
Portal inflammation	Weighted 2x
Hepatocyte apoptosis	Weighted 2x
Centrilobular hypereosinophilia	Weighted 2x
Centrilobular necrosis	Weighted 2x
Microabscesses	Weighted 2x
Circulating neutrophils	Weighted 2x

Supplemental Table 2. Spearman's correlation of tissue cytokine expression and plasma cytokines.

	Spleen Cytokine v Plasma Cytokine		Liver Cytokine v Plasma Cytokine	
	**WT (n=11)**	**Ery$^{tlr9-/-}$ (n=11)**	**WT (n=11)**	**Ery$^{tlr9-/-}$ (n=11)**
**IL-6**	R = 0.936<br>p = 0.00008	R = 0.809<br>p = 0.004	R = 0.909<br>p = 0.0003	R = 0.618<br>p = 0.048
**TNF-α**	R = 0.909<br>p = 0.00027	R = 0.682<br>p = 0.025	R = 0.609<br>p = 0.0519	R = 0.773<br>p = 0.007
**IL-1β**	R = 0.645<br>p = 0.0368	R = 0.118<br>p = 0.735	R = 0.764<br>p = 0.0086	R = 0.005<br>p = 0.995
**IL-10**	R = 0.918<br>p = 0.00019	R = 0.591<br>p = 0.061	R = 0.891<br>p = 0.00052	R = 0.664<br>p = 0.031
**IFN-γ**	R = 0.200<br>p = 0.557	R = 0.291<br>p = 0.818	R = -0.064<br>p = 0.860	R = 0.545<br>p = 0.088

Supplemental Table 3. Primers sequences

Target	Orientation	Sequence (5’ à 3’) or identifier	Use
EpoR Cre	Forward 1	GTGTGGCTGCCCCTTCTGCCA	Genotyping
EpoR Cre	Forward 2	GGCAGCCTGGGCACCTTCAC	Genotyping
EpoR Cre	Reverse	CAGGAATTCAAGCTCAACCTCA	Genotyping
TLR9 flx	Forward	CGGTTAATGGTAGCACTTGG	Genotyping
TLR9 flx	Reverse	GCTTTTGCTCAGAACACAACC	Genotyping
Universal 16S (BSF8)	Forward	AGAGTTGATCCTGGCTCAG	TaqMan qPCR
Universal 16S (BSR357)	Reverse	CTGCTGCCTYCCGTA	TaqMan qPCR
Universal 16S	Probe	/56-FAM/TA A+CA +CAT G+CA +AGT +CGA /3BHQ_1/	TaqMan qPCR
16S multiplex	Forward 1	TCGGMTCGTAAAACTCTGTT	TaqMan qPCR
16S multiplex	Forward 2	GCCTTCGGGTTGTAAAGY	TaqMan qPCR
16S multiplex	Forward 3	GCCTTATGGTTGTAAAGCAC	TaqMan qPCR
16S multiplex	Forward 4	GGTCTTCGGATTGTAAAGC	TaqMan qPCR
16S multiplex	Reverse	CTGCTGGCACGAAGTTAGC	TaqMan qPCR
K. pneumoniae	Probe	/56-FAM/TTAATAACC/ZEN/TYRKCGATTGACGTTACCC/3IABkFQ/	TaqMan qPCR
S. aureus	Probe	/56-FAM/AAGAACATA/ZEN/TGTGTAAGTAACTGTGCACA/3IABkFQ/	TaqMan qPCR
P. aeruginosa	Forward	CTGGAAGCAGGATGGCTATT	TaqMan qPCR
P. aeruginosa	Reverse	CAGTAGCGGGAAGAGAATGTAG	TaqMan qPCR
P. aeruginosa	Probe	/56-FAM/AA CTG CTC T/ZEN/T CCA CCG ACA ACG AC/3IABkFQ/	TaqMan qPCR
18S rRNA	N/A	Hs99999901_s1	TaqMan qPCR
Il6	N/A	Mm00446190_m1	TaqMan qPCR
Tnfa	N/A	Mm00443258_m1	TaqMan qPCR
IL1b	N/A	Mm00434228_m1	TaqMan qPCR
Il10	N/A	Mm01288386_m1	TaqMan qPCR
18S rRNA	Forward	AACCCGTTGAACCCCATT	SYBR green qPCR

18S rRNA	Reverse	CCATCCAATCGGTAGTAGCG	SYBR green qPCR
Il18	Forward	GACAGCCTGTGTTCGAGGATATG	SYBR green qPCR
Il18	Reverse	TGTTCTTACAGGAGAGGGTAGAC	SYBR green qPCR
Ifng	Forward	CAGCAACAGCAAGGCGAAAAAGG	SYBR green qPCR
Ifng	Reverse	TTTCCGCTTCCTGAGGCTGGAT	SYBR green qPCR
